# Frequency of Victimization Experiences and Well-Being Among Online, Offline, and Combined Victims on Social Online Network Sites of German Children and Adolescents

**DOI:** 10.3389/fpubh.2015.00274

**Published:** 2015-12-18

**Authors:** Michael Glüer, Arnold Lohaus

**Affiliations:** ^1^Department of Psychology, Bielefeld University, Bielefeld, Germany

**Keywords:** social online networks, cyberbullying, children and adolescents, self-esteem, self-concept, somatic and psychological symptoms, victimization

## Abstract

Victimization is associated with negative developmental outcomes in childhood and adolescence. However, previous studies have provided mixed results regarding the association between offline and online victimization and indicators of social, psychological, and somatic well-being. In this study, we investigated 1,890 German children and adolescents (grades 5–10, mean age = 13.9; SD = 2.1) with and without offline or online victimization experiences who participated in a social online network (SNS). Online questionnaires were used to assess previous victimization (offline, online, combined, and without), somatic and psychological symptoms, self-esteem, and social self-concept (social competence, resistance to peer influence, esteem by others). In total, 1,362 (72.1%) children and adolescents reported being a member of at least one SNS, and 377 students (28.8%) reported previous victimization. Most children and adolescents had offline victimization experiences (17.5%), whereas 2.7% reported online victimization, and 8.6% reported combined experiences. Girls reported more online and combined victimization, and boys reported more offline victimization. The type of victimization (offline, online, combined) was associated with increased reports of psychological and somatic symptoms, lower self-esteem and esteem by others, and lower resistance to peer influences. The effects were comparable for the groups with offline and online victimization. They were, however, increased in the combined group in comparison to victims with offline experiences alone.

## Introduction

In Germany, about 11% of the children and adolescents in grades 5–10 experience victimization by bullying (1). Bullying is defined as an “aggressive, intentional act carried out by a group or individual, repeatedly and over time against a victim who cannot easily defend him or herself” [(2), p. 376]. Victimization experienced through offline bullying is associated with a number of negative outcomes like somatic and psychological symptoms or reduced wellbeing (3, 4).

### Online Bullying

The definition of online bullying is similar to the definition of offline bullying. It is defined as “an aggressive, intentional act carried out by a group or individual, using electronic forms of contact” [(2), p. 376]. Although online bullying shares some common characteristics with offline bullying like repetition, intention, and power imbalance, there are also important differences [see Ref. (5, 6)]. For example, online bullying compared to offline bullying can be conducted 24 h a day via electronic media. Moreover, perceived anonymity is increased from the perspective of the perpetrator; however, the reward for engaging in online bullying is often delayed (5).

In Germany, the prevalence rates for online victimization vary for children and adolescents aged 6–19 years from 2.9 (7) to 43.1% (8). Some researcher argued that the frequency of victimization may depend on the used media. For German children and adolescents, the prevalence rates for Internet and cell phone use ranged from 5.4 to 7% [age 6–19 years; (9, 10)] and for chat rooms from 4.3 to 43.1% [age 10–19 years; (8)]. A recent representative German study reported that 96% of the children and adolescents aged 12–19 are members of a social online network (SNS) and that most victimization occurred through those communities (11, 12). German children and adolescents reported that they use SNS mostly for sending and receiving messages and for chatting (12). For the German context, little is known so far about the frequency of specific victimization by the use of SNS, although they are the mostly preferred online media in children and adolescents. In a study by Kwan and Skoric (13) of students aged 13–17 using Facebook in Singapore, the most common events of victimization were receiving nasty massages (28.5%) and becoming a laughing stock (23.3%).

### Offline Victimization Versus Online Victimization

A meta-analysis of Kowalski et al. (14) on 137 different data sets suggested that there are higher rates for offline compared to online victimization, and that online victimization is significantly associated with offline victimization. In a German study by Wachs and Wolf (7), with 833 students aged 11–17 years, 11.9% of the students were identified as offline victims and 4% as online victims. Online victimization was also related to offline victimization. In total, 66.2% of online victims were also offline victims. This study contained no information about the frequency of offline and online bullying of children and adolescents participating in a SNS. Kowalski et al. (14) meta-analysis also showed that the association between offline and online victimization was dependent on the country. The association was stronger for North America compared to Europe and Australia, and additionally, the prevalence was slightly higher in North America. This implies that the prevalence of offline, online, and combined victimization depends on the type of media and the country.

Research on online and offline bullying also suggested that victimization varies by sex. The results for sex differences concerning prevalence have been mixed (15). Most studies found that girls are more likely than boys to be victims of online bullying. Other studies have revealed no sex differences concerning online victimization [cf. (14, 16)]. These results differ from findings on offline victimization, where boys are more often victims [i.e., Ref. (17)]. Smith et al. (2) argued that sex differences depend on the kind of media used, such as text messages or SNS. For example, Juvonen and Gross (18) found that girls are more likely victimized by E-Mail and boys by text message. But in a study by Kwan and Skoric (13), children’s gender did not predict the frequency of Facebook victimization. Therefore, it is unclear if there are sex differences regarding SNS victimization in German children and adolescents.

### Associations of Online Victimization to Distress and Self-Concept

Several studies suggested that the negative effects of victimization add up if the bullying incidents occur offline *and* online. In an Austrian study by Gradinger et al. (19), with students aged 14–19 years, victims with combined experiences (offline *and* online victimization) showed more somatic and depressive symptoms compared to offline victims, online victims, or non-involved students. Other studies found additive effects for somatic symptoms, depression, anxiety, stress, aggression, and suicide attempts [i.e., Ref. (20–24)]. The effects of victimization on developmental outcomes can also be moderated by children’s sex. For example, in Kowalski et al. (14) meta-analysis, the effect of victimization on depression was moderated by sex.

Victimization in particular affects the mastering of developmental tasks. Developing a stable, distinct self-concept and high self-esteem are major challenges for adolescents. A problematic self-concept and low self-esteem can lead to adjustment problems, and predicts later depression (25). Victims of offline and online bullying generally have low self-esteem [i.e., Ref. (26); cf. (14)]. According to Hines (27), it can be assumed that social aspects of the self-concept are especially associated with victimization. In his study with children and adolescents aged 11–13 years, offline and online victimization were associated with a reduced self-concept of happiness and satisfaction, increased anxiety and behavior adjustment problems as well as decreased popularity. The meta-analysis of Kowalski et al. (14) showed that the association between online victimization and self-esteem is smaller in European and Australian compared to North American youth.

Although most studies have confirmed the negative additive effect of offline and online victimization, some studies have not. In a study by Olweus (28), effects of victimization on psychological adjustment and well-being were mainly due to offline victimization. In a study by Campbell et al. (20), with students aged 9–19 years, anxiety and depression scores were similar for victims of offline bullying and online bullying. Also, Beckman et al. (29) found no significant differences in psychosomatic problems between online and offline victims. These results are in contrast to the many other studies indicating additive effects.

The above findings show that little is known about the specific victimization effects on children and adolescents participating in SNS. Although many studies reported additive effects of offline and online victimization, there is a need for more detailed information about victimization occurrences in specific media (SNS) and specific countries (such as Germany) and their developmental effects (e.g., on specific parts of the self-concept).

### The Present Study

The research questions of this study focus on the proportion of offline, online, and combined victimization experiences of children and adolescents participating in a SNS. We are interested in the offline, online, and combined victimization prevalences of SNS participants based on a specific filter question that asks for victimization experiences (offline, online, or combined). Based on the filter question, participants were additionally asked to indicate the frequencies of their specific offline and SNS victimization events. It was hypothesized that children and adolescents with combined victimization experiences (based on the filter question) would report more SNS victimization events compared to those who had experienced online or offline victimization alone (Hypothesis 1). This would mean that combined victimization experiences may be regarded as more severe compared to online or offline victimization alone. The confirmation of Hypothesis 1 is an important basis for assuming cumulative risks in the case of combined victimization (offline *and* online). Further, we were interested in the prevalence of specific offline and SNS victimization events in dependency of children’s and adolescents’ sex and their previous victimization experiences (offline, online, or combined). Thus, we additionally want to address the question if girls participating in SNS experience more often online compared to offline victimization.

The main hypothesis is related to the effects of victimization experiences on indicators of social, psychological, and somatic well-being. It is expected (Hypothesis 2) that participants who are victimized (based on the filter question) report more negative outcomes with regard to self-esteem and self-concept, and increased psychological and somatic symptoms. Moreover, it is expected that the effects will be increased for a combination of offline and online victimization. Additionally, we focus on the effects of victimization on specific aspects of the self-concept. We assume that additive effects of victimization can also be shown for specific social parts of the self-concept, such as social competence, resistance to peer influences, and esteem by others. In addition, it will be analyzed if the developmental outcomes of victimization are moderated by children’s sex.

If there are differences in psychological and somatic symptoms, self-esteem and self-concept between the victimization types (offline versus combined/online versus combined), it is further analyzed if these differences are mediated by the frequency of victimization events (Hypothesis 3).

## Materials and Methods

### Procedure and Participants

The study was conducted as a computer-based questionnaire using the EFS Survey software (Version 10.1). The assessments took place in computer labs of participating schools in spring and summer, 2013. The sample consisted of 1,890 children and adolescents in grades 5–10 from 26 German secondary schools. Schools were initially recruited using telephone lists of schools in the region of Northrine-Westfalia (Germany). Eight schools were from two larger cities (100,000–400,000 inhabitants), seven schools were from medium-sized towns (50,000–99,000 inhabitants), and 11 schools were from smaller towns (20,000–49,999 inhabitants). The included schools covered the whole range of performance levels in German secondary schools (11 “Hauptschulen”, 7 “Realschulen”, and 8 “Gymnasien”). In total, 98 classes (26 school classes with 5th graders, 7 with 6th graders, 24 with 7th graders, 17 with 8th graders, 11 with 9th graders, and 13 with 10th graders) participated in the study. There was no difference in victimization type (offline, online, combined, or without victimization experience) or in the frequency of victimization events by school type.

The final analyses were based on the subsample of 1,362 children and adolescents who reported having an SNS account (72.1% of the original sample). In all, 66.2% participated mainly in Facebook, followed by SchuelerVZ^1^ (23.9%), Google+ (19.8%), and Myspace (1.9%). The age range was between 10 and 18 years, with a mean age of 13.9 (SD = 2.1). Information on migration background was not recorded in this study. However, in German secondary schools, 10.1% of the students have a migration background (30).

Participation in the study required parents’ permission. The recruitment of the samples and the study’s procedure were in accordance with the ethical guidelines of the American Psychological Association (APA) and the Society for Research in Child Development (SRCD). The study was approved by an independent ethical review board. Responding to the questions typically took 20–25 min. Different sample sizes in the analyses reported below may be due to missing values in specific measures.

### Measures of Victimization

#### Victimization Types – Filter Question

To identify victims of bullying, a filter question was used for offline, online, combined, or no victimization experiences. Participants were asked if they had previous experience as a victim of bullying. No specific time reference was provided for this question. Possible response categories were *Yes, but exclusively offline*; *Yes, but exclusively online*; *Yes, online and offline*; and *No*. The filter question was accompanied by a detailed explanation of the concept of bullying using Olweus (31) explanation of bullying in his victimization questionnaire. The explanation contained the specific features of bullying (repetition, power imbalance, and intention), with additional bullying and non-bullying examples. Instead of the term *bullying*, the term *mobbing* was used, which is more popular in Germany. Single-item questions were often used in previous research on offline victimization as well as online victimization. In the meta-analysis of Kowalski et al. (14), the effects of single-item and multiple-item measures of online victimization were systematically compared. For most variables included in the meta-analysis (e.g., depression, self-esteem, academic achievement), there was no moderating effect of the kind of measurement on the relation to online victimization. If there was an effect (e.g., for social anxiety), the relationships were smaller for single-item measures. It was also argued (6) that single-item questions may lead to an underestimation of victimization because students may be reluctant to report socially undesirable events. Although a single-item question may underestimate the actual victimization experience, we finally decided to use a single item as a filter question because there were no large differences to the use of multiple-item measures in the meta-analysis of Kowalski et al. (14). In addition, we wanted to build on the self-definition of children and adolescents, and moreover, reduce the amount of questions for the participants. The filter question allowed for the allocation of participants into four groups (no victimization experiences, offline, online, and combined victimization experiences), which is important for the analysis of differences between these groups. Moreover, the filter question provided additional questions that were tailored to the experience of specific offline or online victimization events.

#### Frequency of Victimization Events

After the participants answered the filter question, they answered sets of questions related to their offline, online, or combined victimization experiences. Children and adolescents who reported combined victimization received an online and offline victimization questionnaire, whereas children and adolescents with exclusive offline or online victimization received either the offline or the online questionnaire. The questionnaire for offline victimization comprised a set of seven items with different kinds of victimization events adapted from the Questionnaire on Bullying for Students by Olweus [(31); see Table 3]. The questionnaire for online victimization consisted of 11 items related to different kinds of SNS victimization events adapted from an item set by [Kwan and Skoric (13); see Table 4]. The response scale was for both questionnaires *never*; *only once or twice*; *two or three times a month*; *about once a week*; and *several times a week*. Cronbach’s α was 0.86 for the offline and 0.95 for the online questionnaire.

### Measures of Somatic and Psychological Symptoms, Self-Esteem, and Self-Concept

#### Somatic and Psychological Symptoms

The symptom scales from the German Stress and Coping Questionnaire for Children and Adolescents by Lohaus et al. (32) were included in this study (Table 1). The symptom scales are related to somatic (6 items) and to psychological (12 items) symptoms. All items refer to symptoms experienced during the previous week. A three-point response scale with the labels *never*, *once*, and *more than once* was used.

**Table 1:** **Variables of well-being, self-esteem, and self-concept**.

Instrument	Item examples	Cronbach’s α
**Well-being**		
Somatic psychological symptoms	“How often did you experience a headache last week?”	0.77
Psychological symptoms	“How often did you feel nervous last week?”	0.92
**Self-esteem**	“I always feel very well during leisure time,” “I find myself perfectly okay when I compare myself with my friends”	0.79
**Self-concept**		
Social competence	“I know how to interact with other people”	0.59
Resistance to peer influences	“I state my view even if others think differently”	0.79
Esteem by others	“I have the feeling that others do not want to be friends with me because I am not interesting enough”	0.73

#### Self-Esteem

A scale by Schauder (33) was used to assess self-esteem in this study. The original scale assesses self-esteem in three different contexts (school, leisure/friends, and family), with 18 items each (Table 1). In this study, the subscales related to leisure and friends were used because these topics may be especially important in the context of bullying. Five-point response scales were used, ranging from *I do not agree at all* to *I agree absolutely*.

#### Self-Concept

Three subscales of the Frankfurt self-concept scales by Deusinger (34) were used (Table 1). The subscales are related to (a) social competence, (b) resistance to peer influences, and (c) esteem by others. The social competence scale comprises six items, the resistance to peer influences scale consists of ten items, whereas the esteem by others scale contains six items. A six-point rating scale was applied for the items (1 = *I do not agree at all* to 6 = *I agree absolutely*). The social competence subscale showed a rather low α-value, although a Cronbach’s α of 0.68 was reported in the original study (34). In the present study, the item *I am pretty shy and insecure [when] in contact with others* reached an unsatisfying item-total correlation (<0.3), which may have decreased the α-value. The α-values of all included measures are reported in Table 1.

## Results

### Preliminary Results and Descriptive Statistics

#### Prevalence for Victimization Types

Table 2 contains the prevalences for the different victimization types. The most frequent type was offline victimization. Children and adolescents reported more often combined compared to online victimization alone. As additional analyses showed, the reported victimization types were not related to age, but there was a significant relation to sex (χ^2^ = 21.58, *df* = 3, *p* < 0.001). Girls more often reported victimization online, and combined, compared to boys. Boys reported more offline victimization than did girls.

**Table 2:** **Prevalence of children’s and adolescents’ victimization types (based on a filter question)**.

Victimization type^a^	Participants% (*n*)	Girls% (*n*)	Boys% (*n*)
Total	28.8 (377)	16.7 (219)	12.1 (158)
Offline	17.5 (229)	3.1 (124)	8.0 (105)
Online	2.7 (35)	1.8 (23)	0.9 (12)
Combined	8.6 (113)	5.5 (72)	3.1 (41)

*^a^Percentage is based on 1310 children and adolescents who responded to the victimization types – filter question*.

#### Frequency of Offline and SNS Victimization Events

Tables 3 and 4 contain the means and SDs for the frequency of experienced offline and SNS victimization events (based on the online and offline victimization questionnaires presented after the filter question). Children and adolescents of the combined group report for all events more frequent victimization compared to children and adolesecents with only offline experiences (Table 3). The same applies for the frequency of SNS victimization events (Table 4). However, the mean differences for the frequency of SNS events are much smaller compared to offline victimization events. The most common offline victimizations events were “calling mean names” and “spreading rumors” for both types of victimization (offline and combined). For SNS victimization, the most common events were “receiving nasty messages” and “being blocked” again for both types (online and combined).

**Table 3:** **Means and SDs (in parentheses) of the frequency of offline victimization events for children and adolescents categorized as offline or combined victims (based on a filter question)**.

	Offline victimization types	
	Offline only victims	Combined victims
**Offline victimization events**		
I was called mean names, was made fun of, or teasedin a hurtful way	1.80 (1.21)	2.46 (1.47)
Other students left me out of things on purpose, excluded me from their group of friends, or completely ignored me	1.46 (0.99)	1.84 (1.27)
I was hit, kicked, pushed, shoved around, or locked indoors	1.32 (0.86)	1.42 (1.01)
Other students told lies or spread false rumors about me and tried to make others dislike me	1.76 (1.14)	2.26 (1.40)
I had money or other things taken away from me or damaged	1.26 (0.74)	1.30 (0.78)
I was threatened or forced to do things I didn’t want to do	1.18 (0.63)	1.39 (0.97)
I was bullied with mean names or comments about my race or color	1.41 (0.93)	1.74 (1.06)
Total	1.46 (0.69)	1.78 (0.88)

**Table 4:** **Means and SDs (in parentheses) of the frequency of SNS victimization events for children and adolescents categorized as online or combined victims (based on a filter question)**.

	SNS victimization types	
	Online only victims	Combined victims
**Sns victimization events**		
I have received nasty messages on *Facebook*^a^ which made me upset	1.40 (0.88)	1.91 (1.22)
People have posted messages on *Facebook* about me that damaged my reputation	1.34 (0.91)	1.55 (1.03)
People have said things about me on *Facebook* that caused my friends to dislike me	1.31 (0.90)	1.51 (1.00)
People have said things about me on *Facebook* to make me a laughing stock	1.37 (1.09)	1.59 (1.10)
Someone has hacked into my *Facebook* account and posted/sent messages to make me look bad	1.31 (0.87)	1.32 (0.84)
I have been tricked to share my secret which was later spread on *Facebook*	1.34 (1.06)	1.38 (0.88)
Someone has shared my secrets on *Facebook*	1.31 (1.05)	1.47 (0.97)
I have been blocked on *Facebook* by other people	1.51 (0.95)	1.66 (0.96)
I have been deliberately excluded from a *Facebook* group by people	1.29 (0.86)	1.33 (0.83)
I have received threatening messages on Facebook	1.34 (0.87)	1.47 (0.96)
I have been ignored by my friends on *Facebook* (i.e., received no likes by my friends)	1.26 (0.70)	1.54 (0.99)
Total	1.35 (0.76)	1.54 (0.82)

*^a^The term “*Facebook*” was automatically replaced by the preferred social network site of the child or adolescent*.

#### Intercorrelations Between the Included Variables

As Table 5 shows, there are, in general, negative correlations between the symptom scores and the scores related to self-esteem and self-conception. Moreover, the variables related to self-esteem and self-conception show positive interrelations. Additionally, there are several significant correlations between well-being, the self-related variables, and the measures indicating the frequency of offline and SNS victimization events. The frequency of offline victimization events (based on the offline victimization questionnaire) is related to reduced self-esteem, reduced resistance to peer influences, and reduced esteem by others. The same pattern is associated with combined victimization experiences. The frequency of online victimization (based on the online victimization questionnaire) shows no significant relation to the symptom and self-related scales. However, combined experiences are related to increased somatic symptoms, decreased resistance to peer influences, and decreased esteem by others.

**Table 5:** **Pearson correlations between the variables indicating social, psychological, and somatic well-being and the variables indicating victimization frequency**.

	Indicators of social, psychological, and somatic well-being					Indictors of Victimization			

	Somatic symptoms	Self-esteem	Social competence	Resistance to peer influences	Esteem by others	Frequency of experienced offline victimization events (questionnaire)		Frequency of experienced SNS victimization events (questionnaire)	
						Offline type	Combined type	Online type	Combined type
Psychological symptoms	0.66* (*n* = 1327)^a^	−0.23* (*n* = 1306)	−0.02 (*n* = 1305)	−0.13* (*n* = 1307)	−0.28* (*n* = 1303)	0.06 (*n* = 225)	0.00 (*n* = 111)	−0.07 (*n* = 35)	0.15 (*n* = 110)
Somatic symptoms		−0.23* (*n* = 1306)	−0.01 (*n* = 1305)	−0.15* (*n* = 1307)	−0.28* (*n* = 1303)	0.03 (*n* = 225)	0.14 (*n* = 111)	−0.16 (*n* = 35)	0.20* (*n* = 110)
Self-esteem			0.29* (*n* = 1315)	0.39* (*n* = 1317)	0.49* (*n* = 1312)	−0.21** (*n* = 225)	−0.33** (*n* = 112)	−0.12 (*n* = 34)	−0.18 (*n* = 112)
Social competence				0.27* (*n* = 1341)	0.15* (*n* = 1335)	0.01 (*n* = 225)	−0.13 (*n* = 111)	0.31 (*n* = 35)	0.01 (*n* = 111)
Resistance to peer influences					0.45* (*n* = 1335)	−0.13* (*n* = 224)	−0.24* (*n* = 111)	−0.22 (*n* = 35)	−0.32** (*n* = 111)
Esteem by others						−0.17* (*n* = 224)	−0.18 (*n* = 111)	−0.21 (*n* = 35)	−0.22* (*n* = 111)

*p < 0.05.

**p < 0.01.

*^a^The sample sizes differ because the variables regarding the amount of victimization are related only to the subsamples with victimization experiences; moreover, there are slight variations because of missing data*.

### Differences Regarding the Frequency of Experienced Offline and Online Victimization Events in Dependency of the Victimization Type (Hypothesis 1)

#### Differences Regarding the Frequency of Offline Victimization Events

An analysis of variance was calculated for the frequency of experienced offline victimization events with the independent variables victimization type (offline versus combined) and children’s sex. Due to the high age variance, age was included as a covariate. The results showed significant differences between the children and adolescents with offline and combined experiences [*F*_(1,337)_ = 18.01, *p* < 0.001, η^2^ = 0.05]. Offline victimization events were significantly increased for children and adolescents of the combined group (*M* = 1.78, SD = 0.88) compared to the offline only group (*M* = 1.46, SD = 0.69). Age had a negative effect on victimization [*F*_(1,337)_ = 20.13, *p* < 0.001, η^2^ = 0.06]. Older children reported less victimization. Additionally, there was an effect of children’s sex [*F*_(1,337)_ = 4.44, *p* < 0.05, η^2^ = 0.01]. Boys reported more offline victimization events compared to girls. But there was no interaction between victimization group (offline, online, and combined; based on the filter question) and children’s sex [*F*_(1,337)_ = 1.65, *p* < 0.05, η^2^ = 0.01].

#### Differences Regarding the Frequency of Online Victimization

An analogous analysis of variance for SNS victimization events comparing the online only (*n* = 35) and combined (*n* = 113) victimization group did not show significant differences. There was, however, an effect of children’s age and sex. Older children experienced less SNS victimization events [*F*_(1,143)_ = 9.44, *p* < 0.01, η^2^ = 0.06] compared to younger children, and boys experienced more SNS victimization events compared to girls [*F*_(1,143)_ = 5.73, *p* < 0.05, η^2^ = 0.04]. Again, there was no interaction between victimization group (offline, online, and combined) and children’s sex [*F*_(1,337)_ = 1.65, *p* < 0.05, η^2^ = 0.01].

### Victimization Types and Indicators of Social, Psychological, and Somatic Well-Being (Hypothesis 2)

A multivariate analysis of variance was calculated using the victimization types (without, offline, online, and combined victmization; based on the filter question) and children’s sex as independent variables and age as covariate. Dependent variables were psychological and somatic symptoms, self-esteem, and the self-concept variables (social competence, resistance to peer influences, and esteem by others).

The results showed a significant multivariate effect for victimization type [*F*_(18,3759)_ = 6.45, *p* < 0.001, η^2^ = 0.03]. Moreover, there was a significant multivariate sex effect [*F*_(6,1251)_ = 2.99, *p* < 0.01, η^2^ = 0.01] and an age effect [*F*_(6,1251)_ = 5.08, *p* < 0.001, η^2^ = 0.02]. The univariate analyses for victimization type indicated significant effects for psychological symptoms [*F*_(3,1265)_ = 27.03, *p* < 0.001, η^2^ = 0.06], somatic symptoms [*F*_(3,1265)_ = 18.82, *p* < 0.001, η^2^ = 0.04], self-esteem [*F*_(3,1126)_ = 13.04, *p* < 0.001, η^2^ = 0.03], resistance to peer influences [*F*_(3,1265)_ = 5.32, *p* < 0.01, η^2^ = 0.01], and esteem by others [*F*_(3,1126)_ = 11.21, *p* < 0.001, η^2^ = 0.03]. There were no interaction effects between victimization types and children’s sex [*F*_(18,3759)_ = 0.94, *p* > 0.05, η^2^ < 0.01].

The Bonferroni *post hoc* comparisons indicated that the group without victimization reported less somatic symptoms, less psychological symptoms, more self-esteem, and more esteem by others in comparison to the groups with offline (throughout *p* < 0.001) and combined victimization (throughout *p* < 0.001). Moreover, resistance to peer influences was increased for those without victimization experience compared to the group with combined victimization experiences (*p* < 0.01). The victimization type exclusively online did not differ from the offline and without victimization types. As Table 6 shows, the means of this group are similar to the offline and without victimization groups. Moreover, the group with combined victimization experiences differed from the group with offline only experiences with regard to somatic symptoms (*p* < 0.01) and psychological symptoms (*p* < 0.001). In both cases, the symptom reports were increased for combined victimization experiences. There was, however, no difference between the group with combined experiences compared to the group with online experiences alone.

**Table 6:** **Means and SD (in parentheses) of the variables indicating social, psychological, and somatic well-being by victimization (without, offline, online, and combined; based on a filter question)**.

	No victims	Offline victims	Online victims	Combined victims
Psychological symptoms	1.54 (0.48)^b,c^	1.69 (0.53)^a,c^	1.66 (0.53)	1.92 (0.55)^a,b^
Somatic symptoms	1.60 (0.53)^b,c^	1.78 (0.59)^a,c^	1.77 (0.47)	2.09 (0.60)^a,b^
Self-esteem	3.10 (0.42)^b,c^	2.96 (0.46)^a^	2.89 (0.34)	2.87 (0.47)^a^
Social competence	4.07 (0.86)	4.02 (0.87)	3.67 (0.87)	3.88 (0.89)
Resistance to peer influences	4.54 (0.88)^c^	4.42 (0.94)	4.24 (0.91)	4.21 (0.91)^a^
Esteem by others	4.93 (0.97)^b,c^	4.63 (1.02)^a^	4.77 (0.85)	4.45 (1.03)^a^

*^a^Significant difference in comparison to the non-victims*.

*^b^Significant difference in comparison to the offline victims*.

*^c^Significant difference in comparison to the combined victims*.

The univariate analyses for sex differences indicated an effect for psychological [*F*_(1,1265)_ = 10.02, *p* < 0.01, η^2^ = 0.01] and somatic symptoms [*F*_(1,1265)_ = 17.22, *p* < 0.001, η^2^ = 0.01]. In both cases, girls reported more symptoms compared to boys. Finally, the covariate age was related to self-esteem [*F*_(1,1265)_ = 16.33, *p* < 0.001, η^2^ = 0.01] and resistance to peer influences [*F*_(1,1126)_ = 14.08, *p* < 0.001, η^2^ = 0.01]. Older children showed higher self-esteem and resistance to peer influences.

### The Mediating Effect of Victimization Events (Hypothesis 3)

Since there were differences in somatic and psychological symptoms between the offline only and the combined type a further analysis of a potential mediation through the frequency of offline victimization events can be calculated. According to Baron and Kenny (35), the following four requirements must be met when taking a mediator into account: (1) the predictor (victimization type = offline only and combined) must be associated with the mediator and (2) with the dependent variable (somatic and psychological symptoms). (3) The mediator (frequency of offline victimization events) must be associated with the dependent variable (somatic and psychological symptoms). (4) The direct effect of the predictor (victimization type) must be non-significant in a multiple regression with the mediator. Table 7 shows that the mediator (frequency of offline victimization events) is associated with somatic symptoms but not with psychological symptoms. Therefore, only the mediation for the depended variable somatic symptoms meets all requirements. The multiple regression analysis with somatic symptoms as dependent variable, victimization type (offline only versus combined) as the predictor and frequency of experienced offline victimization events as the mediator showed a significant result [*F*_(2,337)_ = 8.01, *p* < 0.001, *R*^2^ = 0.046]. The regression revealed a significant effect of victimization type (β = 0.18, *p* < 0.01) but no effect of the mediator (β = 0.08, *p* > 0.05). Therefore, the association between victimization type (offline only versus combined) and somatic symptoms was not mediated by the frequency of offline victimization events.

**Table 7:** **Correlations among the predictor, mediator, and dependent variables**.

	Victimization type (1 **=** offline 2 **=** combined)	Somatic symptoms	Psychological symptoms
Frequency of offline victimization events	0.20**	0.12*	0.09
Victimization type (1 = offline, 2 = combined)		0.20**	0.24**
Somatic symptoms			0.66**

*p < 0.05.

**p < 0.01.

## Discussion

This study focused on children and adolescents with an SNS account, which was 72.1% of the original 1,890 participants. For these children and adolescents, the offline and online victimization experiences were compared. It is interesting to note that a large number (28.8%) of participants reported previous victimization. Of those who had experiences as a victim, the prevalence was largest for offline victimization (17.5%) and the combination of offline and online victimization (8.6%). Exclusive online victimization was rarely reported (2.7%). In general, victimization rates vary depending on the measures used (single versus multiple items), provision of a bullying definition, or population characteristics [cf. (14)]. In this study, we investigated German students with an adapted version of the Olweus (31) and Kwan and Skoric (13) questionnaires. For the prevalence rate, we used a single-item measurement approach. We provided a definition of bullying and referred to participants of SNS. We do not know of any other study with the same characteristics to compare our results. Therefore, we can compare only specific study characteristics.

### Prevalence of Victimization Types

The online victimization prevalence of the current study (2.7%) is rather low compared to most German studies, which have reported prevalence rates from 2.9 to 43.1%. A reason may be that the rate of combined victimization (8.6% in this study) increased in recent years because more and more children and adolescents are using the Internet. In total, 11.7% of the participants of this study experienced online victimization (exclusively online and combined). Compared with one of the most recent German studies by Schultze-Krumbholz et al. (10), which investigated online victimization (7%, which may also involve combined victims), the prevalence is rather high for children and adolescent participating in a SNS.

### Differences Between Girls and Boys in Victimization

Concerning sex differences, girls reported more victimization experiences online and combined compared to boys, and boys more often reported offline victimization experiences (based on the filter question). These findings are in line with most previous research (14). The current study also showed that boys reported more offline and SNS victimization events compared to girls when they were asked for specific victimization events. These means, although less boys reported being bullied online (based on the filter question), they experience more online victimization events then girls, if they are bullied. This might indicate that girls and boys not just differ in the prevalence of offline and online victimization but that they also differ in their specific bullying experience. Boys may experience more frequent victimization or may perceive more frequent victimization compared to girls.

### Prevalence of Specific Victimization Events

Facing the specific victimization events, it is interesting to note that for offline victimization the most common events were “calling mean names” and “spreading rumors” for both types of victimization (offline and combined). For SNS victimization, the most common events were “receiving nasty messages” and “being blocked” again for both types (online and combined). “Being blocked” can be interpreted as rather strong form of peer exclusion. Other forms of exclusion like “being ignored” (i.e., receiving no likes) or exclusion in a Facebook group were less frequent. Furthermore, deliberated forms of victimization like hacking somebodies SNS account are rather infrequent.

In general, it was shown (in line with Hypothesis 1) that combined victims differed in their frequency of reported offline victimization events compared to victims with exclusive offline experiences. This emphasizes that combined victimization experiences might lead to more severe offline consequences compared to offline victimization alone. Combined victims seem to be confronted with more stressors, which may increase the risk of negative outcomes in comparison to offline victims. However, this difference could not be shown for the frequency of online victimization events (online versus combined type). This may be due to the small sample size of the group with exclusive online victimization experiences (*n* = 35).

### Victimization Type and Wellbeing, Self-Esteem, and Self-Concept

In line with previous research [cf. (14)] and with Hypothesis 2, the results of this study underline a relationship between victimization type and indicators of social, psychological, and somatic well-being. Children and adolescents without victimization experiences report less psychological and somatic symptoms, higher self-esteem, and more esteem by others compared to participants with offline or combined victimization experiences. These results underline that the victimization type may be a risk factor associated with negative developmental outcomes. Moreover, the current findings show that combined victims also differ significantly from offline only victims with regard to psychological and somatic symptoms. This provides support for a cumulation of risks for the combined victims.

Although previous research has already shown a negative association between online victimization and self-concept, the current study additionally indicates effects on specific parts of the social self-concept. As Table 6 shows especially combined victims are characterized by a lower self-esteem, lower esteem by others, and a reduced resistance to peer influences compared to non-victims. Victims of combined bullying may have more difficulties compared to non-victims in defending their own opinion against others, especially regarding more powerful people. Reduced esteem by others indicates that they may also feel less valued by other people. It is possible that the reduced resistance to peer influences and the lower esteem by others increases their susceptibility to bullying attacks. Victimization, alternatively, may further reduce their resistance to peer influences and their experienced esteem by others.

Victimization was not only associated with effects on self-esteem and self-concept variables, but also on psychological and somatic symptoms. It is known from previous studies that psychological and somatic symptoms are associated with the experience of being bullied (36). The present study underlines this relationship for participants of SNSs. A possible mediator of this relationship may be stress as a consequence of the frequency of specific victimization events. Comparing the different bullying victimization types (offline versus combined/online versus combined) there were only differences in the psychological and somatic symptoms between the offline and the combined group. A mediation analysis revealed that this difference in symptoms are not due to a higher frequency of offline victimization (Hypothesis 3). This leads to the conclusion that the increased psychological symptoms for combined victims may be due to the additional online victimization experience or to other influential factors. Further analyses are needed to analyze possible influential factors and the direction of the association.

### Shortcomings

This study also has several shortcomings: first, a cross-sectional design for data collection was used. Therefore, it is not possible to derive cause–effect relationships from the statistical analyses. Thus, it remains an open question whether the experience of being bullied leads to lower self-esteem, a more negative self-conception, and increased symptoms, or if the opposite causal direction holds: It may also be possible that a low self-esteem or a negative self-conception increases the likelihood of being bullied. Second, a major critical point is the single-item measurement method used. Single-item measures are less time consuming, but single-item measures may underestimate the real number of victims (37). Kowalski et al. (14) has argued that participants may be less willing to answer honestly on single items due to feelings of stigmatization. Nevertheless, in our data set, many participants stated having had victimization experiences. The underestimation effect – if it exists – may affect the association between victimization and outcome variables. In Kowalski et al. (14) meta-analysis, the effect of online victimization on anxiety was higher when multiple-item measurements were used. This may lead to the interpretation that the effects reported in this study may be underestimated.

### Implications

In spite of these shortcomings, the results of the present study nevertheless underline the importance of prevention and intervention to avoid victimization by bullying during childhood and adolescence. They also show the need for a more systematic assessment of different victimization contexts (i.e., SNS), the role of specific input and output factors and the effects in dependency of characteristics like the children’s and adolescents’ sex. Parents, teachers, and especially intervention programs should take such possible differences into account. For example, teachers should be aware of the fact that different media like SNS allow different victimization events and that boys may experience SNS victimization more frequent compared to girls. Of vital importance are the findings of cumulative risks for children with combined victimization experiences. Combined victimization experiences enhance the risk for negative developmental outcomes. As the results show this is especially true for the social parts of the self-concept. More emphasis should therefore be placed to the children’s and adolescents’ self-development when they are confronted with victimization.

## Conflict of Interest Statement

The authors declare that the research was conducted in the absence of any commercial or financial relationships that could be construed as a potential conflict of interest.

## Footnotes

^1^SchuelerVZ was a Facebook-like SNS with approximately three million members in 2008. It closed down in 2013.
